# Checkpoint blockade‐based immunotherapy in the context of tumor microenvironment: Opportunities and challenges

**DOI:** 10.1002/cam4.1722

**Published:** 2018-08-07

**Authors:** Jingjing Duan, Yu Wang, Shunchang Jiao

**Affiliations:** ^1^ School of Medicine Nankai University Tianjin China; ^2^ Department of Oncology General Hospital of Chinese PLA & Beijing Key Laboratory of Cell Engineering & Antibody Beijing China

**Keywords:** biomarkers, checkpoint inhibitors, combination therapy, immunotherapy, tumor microenvironment

## Abstract

A dynamic and mutualistic interaction between tumor cells and tumor microenvironment (TME) promotes the progression and metastasis of solid tumors. Cancer immunotherapy is becoming a major treatment paradigm for a variety of cancers. Although immunotherapy, especially the use of immune checkpoint inhibitors, has achieved clinical success, only a minority of patients exhibits durable responses. Clinical studies directed at identifying appropriate biomarkers and immune profiles that can be used to predict immunotherapy responses are presently being conducted. Combining treatment strategies tailored to cancer‐immune interactions are designed to increase the rate of durable clinical response in patients. It is essential to establish a reasonable tumor classification strategy according to TME to improve cancer immunotherapy. In the current review, a modified classification of TME is proposed, and optimization of TME classification is needed through detailed and integrated molecular characterization of large patient cohorts in the future.

## 1 INTRODUCTION

The successful growth and eventual metastasis of a tumor are not only determined by genetic changes within tumor cells, but also by the adaptive advantage of such genetic alterations in a given tumor microenvironment (TME). Our understanding of the reciprocal communications between cancer cells and tumor stroma has greatly enhanced over the years. Neoplastic tumor cells mediate the recruitment and activation of different stroma cells by releasing specific chemokines. In return, a repertoire of stromal cell types can assist in maintaining the survival advantages of tumors.

Tumors achieve immune privilege through the immunoediting^1^, ^2^, ^3^ of themselves and the formation of an immunosuppressive TME.^4^, ^5^ The heterogeneity of TME can actively shape antitumor immunity and influence therapeutic response.^6^ Co‐inhibitory receptors, such as CTLA4 and PD1, have essential yet distinct^7^ roles in modulating immune responses and have been proven to be valid targets in cancer immunotherapy.^8^, ^9^ Moreover, the next wave of co‐inhibitory receptor targets, including LAG‐3, Tim‐3, and TIGIT,^10^ are being explored in clinical trials. Only a subset of patients, however, exhibit durable responses to an immune checkpoint inhibitor (ICI). Researchers have attempted to improve the effectiveness of checkpoint inhibitors. The first approach involves the careful identification of patients who can potentially benefit from the use of checkpoint inhibitors while sparing others from its high cost and adverse effects. The second approach involves the use of combined onco‐immune strategies, such as chemotherapy and radiotherapy, which can promote the release of tumor antigens, and thus exhibit a synergistic role when combined with immunotherapy.

The state of the TME is heterogeneous, indicating the presence of different immune suppression mechanisms between patients. Hence, only by precise patient selection or tailored onco‐immune therapy can the therapeutic benefits of immunotherapy be maximized. To formulate a more suitable treatment plan for specific TME, we summarize the factors that need to be considered in the evaluation of TME and propose a new immune profile that classifies cancer and guides the checkpoint inhibitor‐based immunotherapy.

## 2 IMPROVING THE EFFICIENCY OF CHECKPOINT BLOCKADE

The response rates to the single‐agent anti‐PD‐L1/PD‐1 antibodies range from 10% to 40%.^11^, ^12^, ^13^, ^14^ Researchers continue to explore methods that will improve the efficiency of immunotherapy. A major hurdle is to predict patient response to immunotherapy. At present, the majority of patients do not respond to immunotherapy; hence, the identification of predictive markers is an area of intense research. Furthermore, the effects of conventional therapies and immunomodulatory drugs on the induction of antitumor immunity need to be further explored to facilitate the formulation of combination strategies.

### 2.1 Patient selection strategies

#### 2.1.1 Biomarkers in tumor cells

##### PD‐L1 expression

The PD‐L1 expression is controversial in determining which individual patient may benefit from anti‐PD‐1/PD‐L1 immunotherapy. Some researchers demonstrate that the expression level of PD‐L1 is significantly associated with better response to anti‐PD‐1/PD‐L1 therapies.^12^, ^14^, ^15^ Several other studies, however, have shown that the efficacy of PD‐1/PD‐L1 inhibitors is independent of PD‐L1 expression.^13^, ^16^ A meta‐analysis that included twenty trials indicated that the response rate in PD‐L1‐positive patients is significantly higher than that of PD‐L1 negative patients.^17^ Notably, however, half of the PD‐L1‐positive tumors were unresponsive, while a significant portion of PD‐L1‐negative patients responded to the PD‐1/PD‐L1 blockade. These findings indicate that the immunohistochemistry (IHC) detection of PD‐L1 is not a perfect biomarker of response to anti‐PD‐1/PD‐L1 immunotherapy. Several reasons account for this. First, the PD‐L1 IHC antibodies used are not uniform in different clinical trials. The 28‐8 clone was used in the nivolumab studies, whereas the SP142 clone was applied in the atezolizumab studies. The Blueprint PD‐L1 IHC assay comparison project^18^ revealed similar performance on tumor cells staining in three assays (22C3, 28‐8, and SP263) and fewer tumor cells staining in the SP142 assay, with low concordance rates in the scoring of immune cells among the four assays. Another comparison study including 493 samples also showed similar patterns of tumor membrane staining in 22C3, 28‐8, and SP263 assays^19^ (Table 1). Second, evaluation criteria and positivity thresholds (from 1% to 50% of tumor cells) used in the PD‐L1 IHC assay vary, with some only calculating tumor cells, whereas others scoring both tumor cells and immune cells. However, no studies have demonstrated a threshold for which the positive or negative predictive value approaches 100%. Third, the expression of PD‐L1 is heterogeneous across patients and within tumors.^20^ Therefore, tumor sampling of one tumor site at one timepoint would not accurately reflect the patient's PD‐1/PD‐L1 expression profile. Lastly, there are different immunosuppressive mechanisms in the TME, and many other factors may affect the efficiency of anti‐PD‐1/PD‐L1 therapy, such as the existence of cytotoxic lymphocytes and the presence of other concurrent immunosuppressive pathways. Hence, the PD‐L1 expression alone may not be enough to evaluate the efficiency of anti‐PD‐1/PD‐L1 therapy.

**Table 1:** The overall percentage of agreement between three assays at multiple tumor expression cutoff levels

Tumors	Patients	Expression cutoff (%)	SP263 vs 28‐8 (%)	22C3 vs 28‐8 (%)	SP263 vs 22C3 (%)	References
NSCLC	39	≥1	89.5	94.7	89.5	18
		≥25	86.8	‐	89.5	
NSCLC	493	≥1	91.7	93.7	91.1	20
		≥10	92.9	94.9	92.7	
		≥25	94.9	96.6	94.3	
		≥50	95.9	97.2	93.5	

##### Mutational or neoantigen burden

The mutational or neoantigen burden has been shown to be a useful predictive biomarker for checkpoint inhibitors. Tumor types with higher mutation load have a better response to immunotherapy.^21^ Melanoma and lung cancer are considered to have the greatest number of neoantigens, so they respond better to checkpoint immunotherapy. The mutational landscape has been shown to shape the response to anti‐PD‐1 therapy in NSCLC.^22^ Higher nonsynonymous mutation burden was more closely associated with pembrolizumab clinical benefit than total exonic mutation burden,^22^ suggesting the importance of neoantigens in dictating the response to immunotherapy. As neoantigen‐specific CD8+ T‐cell responses paralleled tumor regression, it was verified that anti‐PD‐1 therapy could enhance neoantigen‐specific T‐cell reactivity.^22^ Furthermore, a signature defined by mutation‐derived neoepitopes could potentially be used to predict durable clinical benefit from the CTLA‐4 blockade in melanoma.^23^ However, this neoepitope signature was subsequently proven to be not predictive using the same dataset^24^ and in another study of anti‐CTLA‐4 dataset,^25^ indicating the defects of neoantigen peptide as prognostic biomarkers. Importantly, not all tumors with higher nonsynonymous mutations respond to checkpoint blockades,^22^, ^23^ so it is difficult to set a reliable cutoff value. Moreover, the detection of the mutation or neoantigen burden is based on whole‐exome sequencing (WES), which is expensive and time‐consuming, and thus unavailable for broad use in clinical practice. It was reported that cancer gene panels (CGPs) including 315 genes could be used to estimate mutational load and to predict the clinical benefit to the PD‐1 blockade with similar accuracy to that reported using WES.^26^ Noteworthy, predictive accuracy is lost when smaller CGPs (<150 genes) are used. It remains to be explored whether or not CGPs can replace WES for mutational loads detection.

##### Mismatch‐repair (MMR) status

The association of MMR deficiency (dMMR) with the response to checkpoint blockade was firstly demonstrated in colorectal cancer. 50% of patients with dMMR tumors responded to pembrolizumab treatment, while none of the patients with MMR‐proficient tumors responded. Patients with dMMR noncolorectal cancers are also more responsive to checkpoint blockade therapies.^27^ The large proportion of mutant neoantigens in dMMR cancers make them sensitive to immune checkpoint blockade, regardless of the origin of tumor tissues.^28^ Recently, the PD‐1 antibody—pembrolizumab—has been approved for any solid tumor with dMMR, indicating the predictive role of dMMR in response to immune checkpoint blockade therapies.

##### Somatic mutations

Cancer genotypes can determine tumor immunophenotypes and tumor escape mechanisms. In addition to whole‐genome mutation burden, multiple gene alterations have been found to be related to the efficacy of checkpoint inhibitors. In lung adenocarcinoma, TP53 and KRAS mutations were demonstrated to increase PD‐L1 expression, thus facilitating T‐cell infiltration and augmenting tumor immunogenicity. Patients with TP53 and/or KRAS mutations were sensitive to PD‐1 blockade.^29^ Researchers further analyzed the relationship between somatic mutations and the effect of anti‐PD‐1/PD‐L1/CTLA‐4 or other antibodies,^30^ and the results indicated that cancers with specific variations were sensitive to PD‐1 antibodies, while the tumors with EGFR, MDM2, MDM4 and DNMT3A abnormalities were less responsive to PD‐1 antibodies. A high risk of tumor progression was also noted in patients with MDM2 amplification or EGFR mutations when receiving PD‐1 antibody. Furthermore, genomic analysis on a patient with chemorefractory lung cancer who achieved a durable response to anti‐PD‐L1 therapy indicated that variations in JAK3 may contribute to the efficiency of immunotherapy.^31^ Recent studies have identified that specific driver mutations, such as PBRM1 and SMARCA4, may predict the response to PD‐1 blockade in nonhypermutated tumors.^32^, ^33^ Further studies with a large number of samples are needed to establish a definitive role for the use of specific somatic alterations as a mechanism‐based predictive biomarker for immunotherapy.

#### 2.1.2 Biomarkers in immune cells

##### Tumor‐infiltrating lymphocytes (TILs)

High TIL density has been thought to result from a host‐immune response and is considered as an immune‐inflamed phenotype that might serve as a prognostic and predictive biomarker. In the KEYNOTE‐001 study, baseline densities of TILs were quantitatively examined to determine their correlation with the efficiency of pembrolizumab. Results showed that the baseline CD8+ T‐cell density in responders was higher than in nonresponders.^34^ In a Phase 2 study of ipilimumab in metastatic melanoma, however, baseline TIL status was not related to clinical effects.^35^ Instead, the increase in TIL density in tumor biopsy specimens obtained after the second course of treatment with ipilimumab showed a positive association with clinical effects. Another study revealed moderate associations between baseline CD8+, CD3+, and CD45RO+ T‐cell density and response to anti‐PD‐1 treatment, and the associations were more evident after anti‐PD‐1 therapy.^36^ Furthermore, a significantly higher CD8+ TIL density was found in nivolumab responders.^37^ Patients with EBV‐positive gastric cancer who had low mutation burden but stronger evidence of immune infiltration were reported to respond to avelumab.^38^ The baseline density of CD8+ T cells in tumors, however, failed to predict the response to atezolizumab.^16^ Even though more CD8+ TILs were found 6 weeks after onset of durvalumab therapy, it was not associated with clinical activity.^39^ These inconsistent findings and the overlap in baseline CD8+ TILs densities exist in responders and nonresponders make it difficult to establish thresholds as clinical predictive biomarkers for immunotherapy (Table 2).

**Table 2:** Correlation between TILs and the response to immune checkpoint therapy

Agent	Tumors	Collection time	Improved clinical outcome association	References
Pembrolizumab	Melanoma	Pretreatment	Higher CD8+ (but not CD4+) T‐cell densities at the invasive margin and within the tumor parenchyma	34
		On‐treatment	Increase in CD8+ T‐cell density	34
	Melanoma	Pretreatment On‐treatment	A modest association was found between CD8+, CD3+, and CD45RO+ T‐cell densities with clinical benefit. After anti‐PD‐1 treatment, the associations were more significant	36
Nivolumab	NSCLC	Pretreatment	Higher CD8+ TIL density	37
Atezolizumab	Multiple cancers	Pretreatment	Baseline TIL status was not associated with clinical activity	16
Avelumab	EBV‐positive gastric cancer	Pretreatment	Higher lymphocytic infiltration	38
Durvalumab	NSCLC	On‐treatment	More CD8+ TILs during therapy (6 wk after onset of durvalumab therapy) than at baseline was found. However, it was not associated with clinical activity	39
Ipilimumab	Melanoma	Pretreatment	Baseline TIL status was not associated with clinical activity	35
		On‐treatment	Increased TIL density (after the second dose) was associated with significantly greater clinical activity	35

TIL, tumor‐infiltrating lymphocyte; EBV, Epstein‐Barr virus.

##### Immune gene signatures

The immune status of TME can be more widely evaluated through immune gene profiles. A retrospective study of patients with advanced melanoma given ipilimumab demonstrated that the immune gene signatures could potentially serve as effective predictive biomarkers of tumor progression.^40^ Patients with greater pretreatment and posttreatment expression values had longer survival.^40^ Elevated IDO expression and a generalized activation of Th1‐cell response in pretreatment tumors were found to be associated with clinical benefit.^16^ Another analysis revealed that high expression of HLA‐DR, an MHC class II molecule, was associated with increased clinical response to anti‐PD‐1/PD‐L1 therapy.^41^ Interferon‐γ (IFN‐γ), an important regulator of immune response, is released by activated T cells and upregulates PD‐L1. Melanoma patients that responded to anti‐PD‐L1 treatment had elevated baseline serum IFN‐γ expression, as well as IFN‐γ‐inducible gene expression.^16^ The expression of both IFN‐γ‐10 and expanded immune‐28 genes were significantly associated with clinical response in the KEYNOTE‐001 trial.^42^ Another study showed that the IFN‐γ‐responsive gene expression profiles were necessary, but not always sufficient, for clinical benefit of PD‐1 blockade.^43^ The correlation between immune gene signatures and response to checkpoint blockade needs more exploration.

##### T‐cell receptor (TCR) clonality

The next‐generation sequencing was used to identify all the uniquely rearranged variable β‐chain regions in TCR. Tumeh et al^34^ investigated whether the narrow TCR repertoire in tumors correlated with the response to pembrolizumab and found that patients with more clonal restricted and less β‐chain diversity were sensitive to pembrolizumab. Compared to the progression group, these clones increased 10‐fold in the responding group after the anti‐PD‐1 treatment, indicating a tumor‐specific response. However, the relevant data are limited, and the way to match tumor antigen and TCR repertoire has not been determined.

##### Peripheral immune‐inflammatory cells and cytokines

Researchers are exploring the potential of peripheral blood markers to serve as noninvasive biomarkers in patients receiving immune checkpoint therapies. At baseline levels, markers such as low neutrophil count, low neutrophil/lymphocyte ratio, and high frequency of lymphocyte have been associated with the response to ipilimumab.^44^ Changes related to ipilimumab‐induced clinical benefit also include decreased frequency of Treg cells and increased lymphocytes and eosinophils.^45^ The magnitude of the reinvigoration of circulating exhausted phenotype T cells in relation to pretreatment tumor burden was also found to be correlated with clinical response.^46^

Serum cytokine levels have been investigated to predict clinical benefit of immune checkpoint blockade. Changes in serum IL‐8 levels could monitor and predict clinical response of immune checkpoint blockade in melanoma and NSCLC patients.^47^ Early changes in serum IL‐8 levels (2‐4 weeks after treatment initiation) were strongly associated with response to checkpoint blockade treatment, with decreased IL‐8 levels in responders and increased IL‐8 levels upon cancer progression. More clinical studies are needed to confirm the predictive value of IL‐8 in the clinical benefit of immune checkpoint blockade treatment.

#### 2.1.3 Gut microbiota

Preclinical studies in mice have highlighted the critical role of gut microbiota in mediating tumor responses to chemotherapeutic agents^48^, ^49^, ^50^ and immunotherapies targeting PD‐L1 or CTLA‐4.^51^, ^52^, ^53^, ^54^ The diverse types and variable distribution of gut microbes affect the degree of T‐cell infiltration into tumors, which is closely related to natural antitumor responses. Commensal bacteria will penetrate into the gut lamina propria and cause dysbiosis following chemotherapy, which activates the host's innate immune system and facilitates the induction of antitumor immunity. Mice with subcutaneous syngeneic tumors do not respond to chemotherapeutic drugs if they receive prior treatment with antibiotics or when they are raised in germ‐free conditions.^49^, ^50^ *Enterococcus hirae* and *Barnesiella intestinihominis* were reported to be involved in response to the immunomodulatory agent cyclophosphamide (CTX). *E. hirae* translocated from the small intestine to secondary lymphoid organs and increased the intratumoral CD8/Treg ratio, whereas *B. intestinihominis* accumulated in the colon and promoted the infiltration of IFN‐γ‐producing γδT cells in cancer lesions, thus facilitating CTX‐induced immunomodulatory effects.^55^ Researchers found that the initial resistance to ICIs can be attributed to abnormal gut microbiome composition through different mechanisms. Metagenomics of patient stool samples revealed correlations between responses to ICIs and the relative abundance of *Akkermansia muciniphila* 51 and the *Ruminococcaceae* family.^52^ Oral supplementation with *A. muciniphila* restored the efficacy of PD‐1 blockade in an IL‐12‐dependent manner by increasing the recruitment of CCR9+ CXCR3+ CD4+ T lymphocytes.^51^ *Bifidobacterium* was also found to be associated with the antitumor effects, and the combination of oral administration of *Bifidobacterium* and PD‐L1 blockade nearly abolished tumor outgrowth.^54^ Augmented dendritic cell function leading to enhanced CD8 T‐cell priming and accumulation in the TME mediated this effect.^54^ Furthermore, increased representation of bacteria belonging to the *Bacteroidetes phylum* was correlated with resistance to the development of checkpoint blockade‐induced colitis.^56^ Overall, it is unclear which bacterial species are involved in tumor immunosurveillance and how the microbiome influences the host response to immunotherapies. Thus, it remains an intensive area of research.

#### 2.1.4 Dynamic biomarker

Biomarker studies (Table 3) have focused on pretreatment characteristics. However, tumor‐bearing inbred mice with identical germline genomes show differences in their response to checkpoint blockade,^57^ suggesting that pretreatment condition cannot fully explain the host response to checkpoint blockade. Therefore, whether the therapies can work or not may be partly decided after the therapy has been administered followed by the critical changes in TME. Chen et al^36^ studied a cohort of melanoma patients treated with checkpoint inhibitors and analyzed immune signatures in longitudinal tissue samples collected at multiple time points during the therapy. The results indicated that adaptive immune signatures in early treatment tumor biopsy samples, rather than the pretreatment patterns, are highly predictive of the response to checkpoint blockade, suggesting repeated biopsies may be needed in further investigations to determine the immune profile in response to immunotherapies with accuracy.

**Table 3:** Predictive biomarker strategies under development for checkpoint immunotherapy

Predictive biomarkers	Details of approach	Improved clinical outcome association	Sample	Sample collection time	Challenges for application in the clinic
Tumor cell					
PD‐L1 expression	IHC‐based evaluation of PD‐L1‐positive tumor cells or immune cells, or both	Positive PD‐L1 tumor status	Tissues	Pretreatment	The dynamic and focal nature of PD‐L1 expression makes it difficult to determine the actual status of the PD‐1/PD‐L1 axis
Mutational burden	NGS‐based (WES/CGP) assessment to calculate the nonsynonymous mutations	Higher nonsynonymous mutation burden	Tissues	Pretreatment	A reliable cutoff value has not been set
Neoantigen burden	WES‐based prediction of neoantigens	Higher predicted neoantigen signature	Tissues	Pretreatment	A reliable cutoff value has not been set
Mismatch‐repair status	NGS‐based or IHC‐based	MSI‐H or Mismatch‐repair deficiency	Tissues	Pretreatment	Approved by FDA
Somatic mutations	NGS‐based	Defined somatic gene mutations, such as TP53, KRAS, JAK3, and POLE	Tissues	Pretreatment	Underinvestigated and more clinical studies are needed
Immune cell					
Tumor‐infiltrating lymphocytes	IHC‐based assessment of the invasion of T cells at tumor bed	Increased CD8+ tumor‐infiltrating lymphocyte density	Tissues	Pretreatment On‐treatment	A reliable cutoff value has not been set
Immune gene signatures	Assessment of gene expression using an automated platform	Interferon γ‐inducible signatures or T‐cell‐inflamed profile	Tissues	Pretreatment	More clinical studies are needed
T‐cell receptor clonality	NGS‐based assessment of T‐cell receptor β chain	More clonal restricted and less β‐chain diversity of T cells in tumor	Tissues	Pretreatment	More clinical studies are needed
Peripheral immune‐inflammatory cells	Hematological examination or flow cytometry	More immune effector cells and fewer immunosuppressive cells	Peripheral blood	Pretreatment On‐treatment	Underinvestigated and more studies are needed
Peripheral cytokines	ELISA‐based	Decreased serum IL‐8 levels (2‐4 wk after treatment initiation)	Peripheral blood	Pretreatment On‐treatment	More clinical trials are needed to investigate the role of IL‐8 and others
Gut microbiota	16S ribosomal RNA gene sequencing‐based	Defined species of gut bacteria, such as *A. muciniphila* and the *Ruminococcaceae* family	Fecal	Pretreatment	Gut microbiota is more complicated than we have explored, more basic studies and clinical research are needed.
Dynamic biomarker strategy	Multiple approaches	Adaptive immune signatures in early treatment tumor biopsy samples	Multiple samples	Pretreatment On‐treatment	Multiple biopsies are of significant challenges in clinic

IHC, immunohistochemistry; NGS, next‐generation sequencing; WES, whole‐exome sequencing; CGP, cancer gene panel; ELISA, enzyme‐linked immunosorbent assay.

### 2.2 Onco‐immune combination therapies

An improved understanding of cancer‐immune interactions has increased the number of patients benefiting from immunotherapy. The goal of combination immunotherapy is to produce a durable antitumor response in patients who do not benefit from monotherapy. Several combination strategies have already been proposed.^58^, ^59^, ^60^, ^61^

The mechanisms of immune checkpoints blockade support the rational design of their combinations in cancer immunotherapy.^62^ Clinical trials by far have verified the favorable objective response rate of the combination of the PD‐1/PD‐L1 blockade and CTLA‐4 blockade in patients with lung cancer^63^, ^64^, ^65^, ^66^ and melanoma.^67^, ^68^, ^69^, ^70^ Nivolumab plus ipilimumab showed manageable safety profiles in CheckMate 032.^66^ Grade 3 or 4 treatment‐related adverse events, most commonly being increased lipase and diarrhea, occurred in 13% of patients in the nivolumab monotherapy cohort, and 30% in the nivolumab (1 mg/kg) plus ipilimumab (3 mg/kg) cohort. In CheckMate 067, treatment‐related adverse events of grade 3 or 4 occurred in 59% of the patients in the nivolumab‐plus‐ipilimumab group and in 21% or 28% of those in the nivolumab or ipilimumab group, respectively. The most common treatment‐related grade 3‐4 adverse events in the combination group were colitis and increased alanine aminotransferase. The toxicity of the concurrent therapy with nivolumab‐plus‐ipilimumab was higher but remained manageable. It is worth noted that fatal myocarditis occurred in the combined application cases due to an overactive immune response,^71^ suggesting that treatment‐related toxicity should be carefully monitored when two checkpoint inhibitors are used together.

A large number of clinical trials are also being conducted on the use of a combination of ICIs and radiotherapy or chemotherapy. Radiotherapy modulates the antitumor immune response through a variety of ways,^72^ such as increasing the release of tumor antigens, enhancing the function of antigen processing cells,^73^ or enhancing T‐cell effector activity.^74^ Evidence from early phase clinical trials evaluating the efficacy of the combination of radiotherapy and immunotherapy has demonstrated therapeutic benefits compared to the use of either therapy alone.^75^ Similar to radiotherapy, chemotherapeutic agents elicit the release of tumor antigens or exhibit immunostimulatory functions^76^ particularly through the regulation of the effector function of macrophages.^77^ Clinical trials show that the addition of immunotherapeutic agents to chemotherapy is predicted to enhance and synergistically increase the antitumor effects of either treatment modality alone.^78^ Despite these promising outcomes, the sequencing and dosing of radiotherapy or chemotherapy in combination with immunotherapy that provides optimal therapeutic benefit have not been rationally investigated.

Targeted inhibitors of various oncogenic signaling pathways have yielded promising therapeutic benefit in some cancers. Given that some of these agents have been shown to modulate cancer‐immune interactions, the combination of immunotherapy and targeted therapies will probably be a more effective strategy in cancer treatment than monotherapy. For example, the combination of immunotherapy and anti‐angiogenesis therapy has been explored from bench to bedside to treat cancer. The initiation of angiogenesis and immunosuppressive responses is part of a physiological and homeostatic tissue repair program, which can be co‐opted in pathological states, notably by tumors. Within the TME, various cell types with established roles in immunosuppression have been shown to boost angiogenesis through the production of multiple growth factors. In return, immune cell recruitment and immune response can be directly modulated by endothelial cells. The disruption of vessel normalization reduced T lymphocyte infiltration, and the depletion or inactivation of T lymphocytes decreased vessel normalization in return, indicating the reciprocal loop between angiogenesis and immunosuppression^79^; thus, combination therapy is theoretically promising. Preclinical studies indicated that a combined angiopoietin‐2 and VEGFA blockade by a bispecific antibody (A2V) inhibits tumor angiogenesis, normalizes blood vessels, activates immune cells, and promotes the extravasation and perivascular accumulation of T cells in tumors.^80^ PD‐1 blockade enhances the antitumor activity of A2V, and the antitumor activity of A2V is CTL‐dependent. Anti‐PD‐L1 therapy could sensitize tumors to anti‐angiogenesis therapy and prolong its efficacy. Meanwhile, anti‐angiogenesis therapy can improve the effects of anti‐PD‐L1 treatment especially when it generates high endothelial venules that facilitate enhanced CTL infiltration and activity.^81^ The combination of bevacizumab and ipilimumab was reported to achieve a favorable response and can be safely administered together in patients with melanoma.^82^ Many more clinical trials of onco‐immune combination therapies are ongoing to provide a more effective strategy in cancer treatment.

Furthermore, epigenetic therapies, such as histone deacetylase inhibitors or DNA methylation inhibitors, have potential to render cancer cells more immunogenic. Thus, combining immunotherapy with epigenetic drugs has been proposed. Numerous immunomodulatory pathways, as well as inhibitory factors, are potential targets for synergizing with immune checkpoint blockade.^61^ The combination of different immunotherapy approaches is currently being explored. The presence of an immunosuppressive microenvironment can limit the full potential of adoptive T‐cell immunotherapy. Hence, specific blockade of the PD‐1 pathway can enhance the function of gene‐modified T cells.^83^ CAR‐T cells expressing a PD‐1‐dominant negative receptor were reported to provide cell‐intrinsic checkpoint blockade and augment antitumor efficacy.^83^ The combination of CAR‐T therapy and checkpoint blockade is under investigation.

## 3 IMMUNOTHERAPY‐BASED TREATMENT SHOULD BE TAILORED TO TME

A critical question in immunotherapy is to choose a tailored single‐agent or combination immunotherapy regimen for a particular patient. A variety of factors contribute to the timing of the immunotherapy‐induced antitumor immune response. Specific immune phenotypes can help set a framework to identify pathways that elicit the best response for each tumor type. Thus far, two different immune profiles have been proposed to classify immunotherapy‐induced response. One consists of four groups based on the PD‐L1 status and the presence of T‐cell infiltration.^84^ The other classification is based on the spatial distribution of TILs and comprises immune‐inflamed, immune‐excluded and immune‐desert phenotypes.^85^ Both of the classification systems distinguish between different immune tolerance mechanisms in the TME, and each system has its advantages. The former profile considers PD‐L1 status as the unprecedented number of durable clinical responses to anti‐PD‐1/PD‐L1 therapies obtained in various cancer types, whereas based on the spatial distribution of T‐cell infiltration, the latter accords with the mechanism of the cancer‐immune cycle.

**Figure 1:**
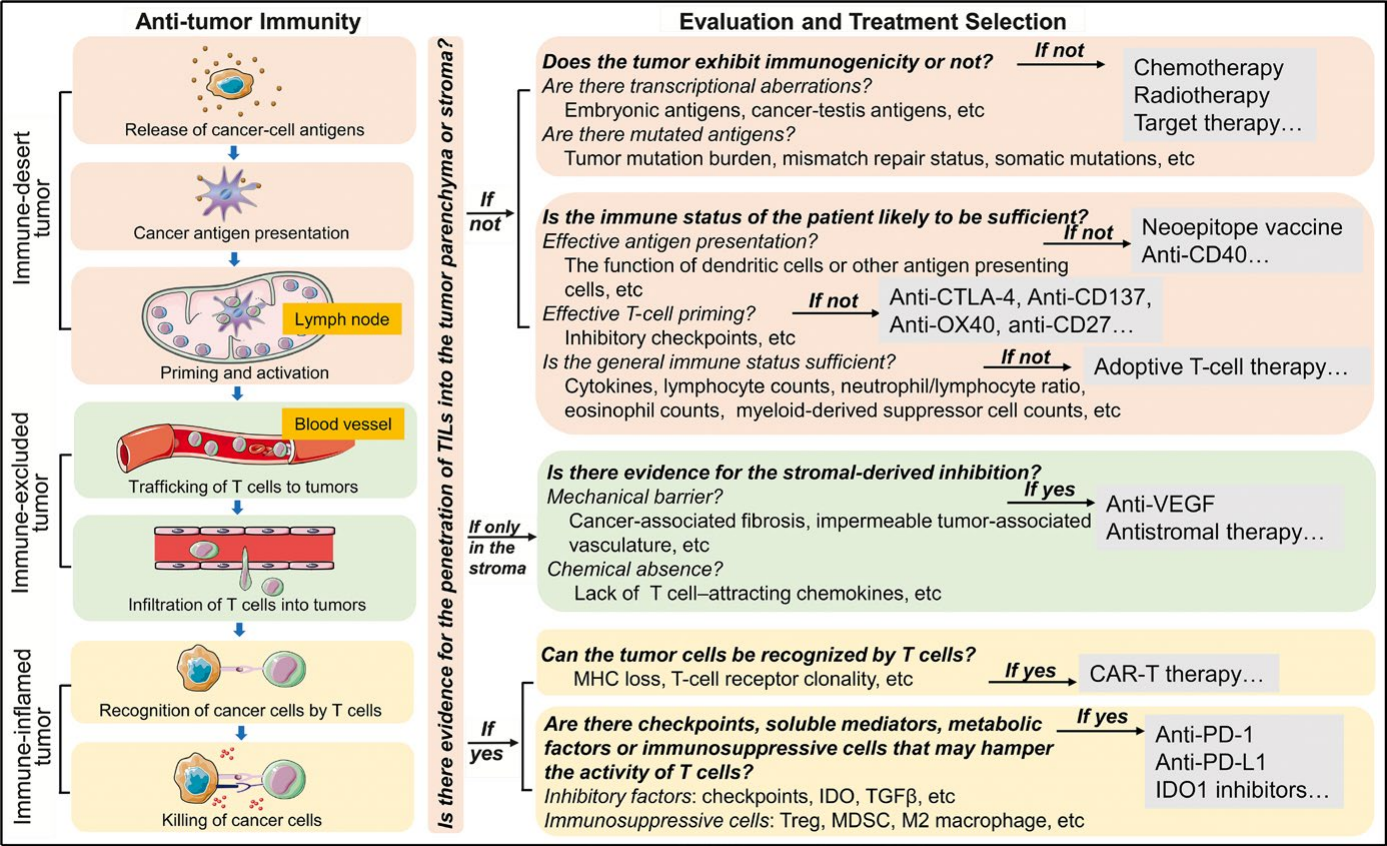
The evaluation of the cancer‐immune interactions. A series of different steps, called cancer‐immunity cycles, have been proposed. The tumor microenvironment can be divided into three phenotypes according to the cancer‐immune interactions. The immune‐desert phenotype, characterized by a paucity of T cells in either tumor parenchyma or the stroma, results from the absence of immunogenicity, or a lack of appropriate T‐cell priming or activation. In the immune‐excluded tumors, the immune cells cannot penetrate the tumor parenchyma but instead are retained in the stroma, reflecting a specific chemokine state or the presence of particular vascular barriers. Immune‐inflamed tumors are characterized by the infiltration of various subtypes of immune cells, including immune‐activated and immune‐inhibitory cells; the immune cells are positioned in proximity to the tumor cells, indicating that a preexisting antitumor immune response is arrested. Each phenotype is associated with specific underlying mechanisms that may prevent the host's immune response from eradicating cancer. Hence, each step in the cancer‐immunity cycle should be carefully evaluated to determine which inhibitory factor is dominant, thus guiding the selection of precise therapies accordingly

As shown in Figure 1, each step in the cancer‐immunity cycle can block the immune response; thus, careful evaluation is necessary to dissect the immune response in cancer. The first and the most critical step of immune response evaluation is to assess the penetration of TILs into the tumor site. According to the spatial distribution of TILs, we can distinguish between the three different phenotypes. In the immune‐desert tumors, the generation of tumor‐specific T cells is the rate‐limiting step of antitumor response. The tumor immunogenicity or neoantigen epitope should be estimated to determine whether the tumor exhibits immunogenicity or not. Traditional therapies, such as chemotherapy and radiotherapy, are recommended for tumors with low immunogenicity to promote the release of cancer‐associated antigens. Specific TCR‐T cells are designed to identify particular neoantigen epitope in tumors. Then, we need to consider whether the immune status of the patient likely to be sufficient to complete antigen presentation and achieve the priming and activation of T cells? If not, the following strategies can be applied to activate T cells: The neoepitope vaccine can be applied to overcome defects in antigen presentation; anti‐CTLA4 antibodies can help activate T cells; and adoptive T‐cell therapy will be a choice to the immune‐desert tumor type. As specific mechanical or chemical barrier exists in the immune‐excluded tumors, anti‐angiogenesis or particular antistromal therapy should be considered. For the immune‐inflamed phenotype, the block lies in the last two steps in the cancer‐immunity cycle. We should figure out whether the tumor cells can be recognized by T cells and whether inhibitory checkpoints and immunosuppressive cells that may hamper the activity of T cells exist. These evaluations can help an oncologist come up with treatment options in a more refined and personalized manner.

**Figure 2:**
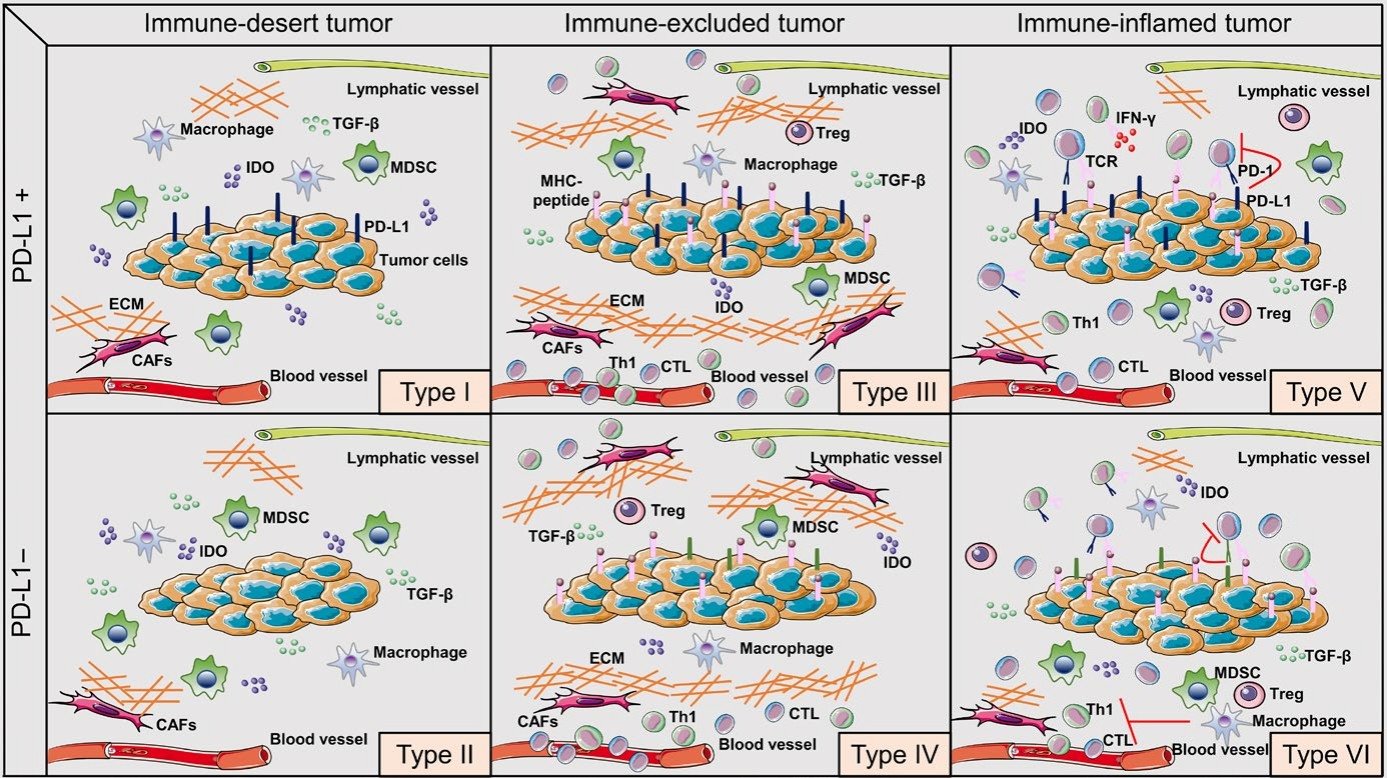
Modified immune profiles. Cancers have been categorized into six different tumor microenvironments based on the spatial distribution of lymphocytes and the expression status of PD‐L1. The dominant immunosuppressive mechanisms are significantly different in the various immune profiles. The categories are Type I (PD‐L1 positive in immune‐desert tumors), Type II (PD‐L1 negative in immune‐desert tumors), Type III (PD‐L1 positive in immune‐excluded tumors), Type IV (PD‐L1 negative in immune‐excluded tumors), Type V (PD‐L1 positive in immune‐inflamed tumors), and Type VI (PD‐L1 negative in immune‐inflamed tumors). Th1, T helper 1; CTL, cytotoxic T lymphocytes; Treg, regulatory T cells; MDSC, myeloid‐derived suppressor cells; CAFs, cancer‐associated fibroblasts; ECM, extracellular matrix

However, such an evaluation of the cancer‐immunity cycle is much too complicated. Therefore, the simple initial stratification of TME may be a favorable choice. As PD‐1/PD‐L1 blockade is considered a broad‐spectrum anticancer therapy at present and the spatial distribution of TILs can represent the status of the cancer‐immune interactions to some extent, we combined the two aforementioned immune classifications into an integrated whole. As shown in Figure 2, the combined immune profile comprises six phenotypes, including Type I (PD‐L1 positive in immune‐desert tumors indicating intrinsic induction), Type II (PD‐L1 negative in immune‐desert tumors indicating immune tolerance), Type III (PD‐L1 positive in immune‐excluded tumors indicating intrinsic induction of PD‐L1 accompanied by stromal‐based immunosuppression), Type IV (PD‐L1 negative in immune‐excluded tumors indicating stromal‐based inhibition), Type V (PD‐L1 positive in immune‐inflamed tumors driving adaptive immune resistance), and Type VI (PD‐L1 negative in immune‐inflamed tumors indicating the role of other suppressor pathways in promoting immune ignorance).

**Figure 3:**
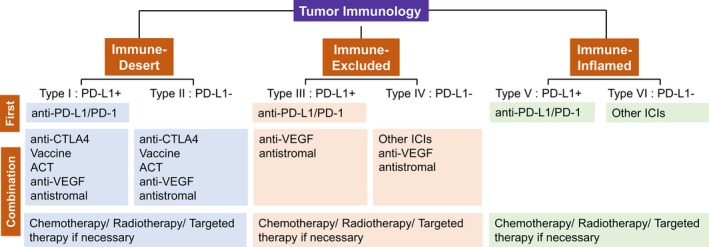
Modified immune profiles for tailoring immunotherapy‐based treatment. The presented modified framework for stratifying tumors is simplistic but allows a platform to discuss the immune‐based therapies best suited to the six different tumor microenvironments. Generation of tumor‐specific T cells is the rate‐limiting step in Type I and Type II tumors, and thus, combination therapy designed to activate T cells, bring specific T cells into tumors and then avoid them being turned off, would be considered. T‐cell migration through the tumor stroma is the rate‐limiting step in Type III and Type IV tumors; thus, antistromal therapy is recommended to break the mechanical barrier. As the inflamed environment facilitates the antitumor immunity, and the preexisting antitumor immune response is turned off by the particular checkpoint or other suppressors, therapies targeting specific checkpoint or other suppressors may be the priority in Type V and Type VI tumors. ACT, adoptive cell therapy. ICIs, immune checkpoint inhibitors

This combined classification can help make the preliminary judgment on the primary immune tolerance mechanism present in particular patients (Figure 3). Briefly, Type I, Type III, and Type V are all PD‐L1‐positive tumors; hence, anti‐PD‐1/PD‐L1 therapies may be the priority of immunotherapy targeting these types of tumors. Type I tumors lack T‐cell infiltration due to the low immunogenicity of tumor cells or impaired antigen presentation and thus may be given radiotherapy to induce cell death and liberate neoantigens, or therapies increase T‐cell numbers and activate T‐cell functions. Besides, the stromal inhibition might coexist in the “immune‐desert” tumors, in which the inclusion of anti‐VEGF or antistromal therapies could be crucial. While in Type III tumors, a preexisting antitumor response might have been present but was rendered ineffective by a block of T cells penetrating through the stroma. After treatment with single‐agent anti‐PD‐1/PD‐L1 antibodies, stroma‐associated T cells can show evidence of activation and proliferation but not infiltration; thus, clinical responses are uncommon. Therefore, these tumors may simultaneously receive anti‐angiogenesis or specific antistromal therapies to facilitate the infiltration of T cells. Type I and Type III tumors can explain why some PD‐L1‐positive tumors cannot benefit from anti‐PD‐1/PD‐L1 monotherapy. In contrast, Type V tumors may benefit from single‐agent anti‐PD‐1/L1 blockade, as these tumors have preexisting T cells that are turned off by PD‐L1 engagement. Hence, the correct identification of tumor subtype may allow specific patient populations benefit from single‐agent checkpoint inhibitors while avoiding high costs and adverse effects from using combined therapies. For Type II tumors, combined therapy would be designed to activate T cells, bring specific T cells into tumors, and then avoid them being turned off. The combination of anti‐CTLA‐4 and anti‐PD‐1 was shown to be useful both in patients with PD‐L1‐positive and PD‐L1‐negative tumors. Therefore, combined checkpoint blockade treatment could be used against the immune ignorance of Type II tumors. Anti‐angiogenesis or specific antistromal therapies may facilitate the infiltration of T cells into Type IV tumors. Other suppressive pathways might be dominant in Type VI tumors; thus, targeting other non‐PD‐1/PD‐L1 checkpoint receptors or other immunosuppressive pathways, such as IDO, may be amenable in treating Type VI tumors. The combination of immunotherapy and traditional antitumor therapies was considered in all six types if necessary, as chemotherapy/radiotherapy/targeted therapies not only remain the first‐line treatment for many cancers but also can induce immunomodulatory effects through diverse mechanisms, and favorable results were shown in clinical trials regarding these combination strategies.

There are several limitations of our model. First, the spatial distribution of TILs together with tumor PD‐L1 status may not necessarily decide whether therapeutic intervention can reactivate tumor‐specific T cells; instead, tumor genetics and the status of other inhibitory factors will probably contribute to tumor‐specific T‐cell activation. Second, given tumor heterogeneity and the focal nature of PD‐L1 expression within many tumors, the biopsy may lead to a false‐negative result if fine needle specimens or cytological specimens are evaluated. Besides, the expression of PD‐L1 is dynamic especially during the treatment, and thus, a static image of one or few biopsies may not accurately reflect the potential complexity of TME or predict response to therapy. A dynamic reevaluation of the TME state at the appropriate time is necessary and can guide the tumor treatment more accurately. Lastly, most of the clinical trials considered the higher tumor PD‐L1 expression to be a priority of ICI use. Our model only takes the expression of PD‐L1 on the tumor cells into account and will need optimization when more evidence on the PD‐L1 expression on specific stroma cells appear. Nevertheless, based on the present knowledge, the expression of PD‐L1 may also be therapeutically relevant and must be carefully considered in the stratification of tumor types. With further understanding of tumor immune microenvironment, this model needs more optimization to improve TME classification and guide cancer immunotherapy.

## 4 FUTURE CHALLENGES

Despite the success in targeting tumor stroma, tremendous challenges still lie ahead. The challenges are summarized in three aspects as follows.

There are various components in the TME; hence, it is difficult to fully understand the mechanism of how a particular TME leads to immunosuppression as a whole. We need to determine whether or not the characteristics of TME are identical in the primary and metastatic sites and identify which one or more components account for the most of TME‐induced immunosuppression under specific conditions. Different parts of the TME may play immune‐inhibitory roles synergistically; hence, exploring the immunosuppression mechanism caused by a single component is limited and targeting only one facet of cancer biology may fail when applied to the general population.

In the shadow of substantial tumor heterogeneity, another major challenge is to assess the TME with precision. Reliable biomarkers that can be utilized to evaluate the TME are currently not available. Intra‐ and intertumor heterogeneity and the dynamic change of cancer‐immunity cycles can explain the difficulty in identifying predictive biomarkers. The immune classification of TME that we modified could partially elucidate the heterogeneity in tumors and efficiently stratify patients into different categories. However, more elaborate biomarkers should be taken into account to perform the precise patient selection.

Additionally, the timing to re‐evaluate the TME to determine drug response is not known. Tumor cells and TME are in continuous dynamic cross talk during therapy, so it is equivocal whether baseline characteristics or posttreatment features can better represent drug response. Multiple biopsy samplings over time and space are needed to track the dynamic changes of drug response.

Precise patient selection and tailored combination strategies may offer an opportunity to conquer cancer; however, assessing TME accurately and elucidating the relationship between TME and therapeutic response remain a significant challenge.

## CONFLICT OF INTEREST

The authors made no disclosures.
